# Acute Caffeine Ingestion, Calendar-Based Menstrual-Cycle Window, Time of Day, and Match-Induced Fatigue Independently and Interactively Influence Psychophysiological, Cognitive, and Physical Performance in Elite Female Volleyball Players: A Randomized Double-Blind Placebo-Controlled Crossover Design Study

**DOI:** 10.3390/life16060922

**Published:** 2026-05-30

**Authors:** Meher Seddik, Wissem Dhahbi, Manel Bessifi, Imen Moussa-Chamari, Halil İbrahim Ceylan, Nagihan Burçak Ceylan, Raul Ioan Muntean, Dražen Čular, Nizar Souissi

**Affiliations:** 1 Physical Activity, Sport and Health Research Unit (UR18JS01), National Observatory of Sports, Tunis 1003, Tunisian_souissi@yahoo.fr (N.S.); 2 Research Unit (UR22JS01) “Sport Sciences, Health and Movement”, High Institute of Sport and Physical Education of Kef, University of Jendouba, El Kef 7100, Tunisiamanelbessifii@gmail.com (M.B.); 3 Training Department, Police College, Qatar Police Academy, Doha 7157, Qatar; 4 Sport Coaching Department, College of Sport Sciences, Qatar University, Doha P.O. Box 2713, Qatar; 5 Physical Education of Sports Teaching Department, Faculty of Sports Sciences, Atatürk University, 25240 Erzurum, Türkiye; 6 Graduate Education Institute, Bayburt University, 69000 Bayburt, Türkiye; 7 Department of Physical Education and Sport, Faculty of Law and Social Sciences, University “1Decembrie 1918” of Alba Iulia, 510009 Alba Iulia, Romania; 8 Faculty of Kinesiology, University of Split, 21000 Split, Croatia; 9 European Institute for Talents, Education, Research & Development, 21000 Split, Croatia; 10 High Institute of Sport and Physical Education, University of Manouba, Ksar-Said, Mannouba 2010, Tunisia

**Keywords:** adenosine receptor antagonist, chronobiology, countermovement jump, executive function, neuromuscular fatigue, ovarian hormones, psychomotor performance, team-sport athletes

## Abstract

**Aim:** Female athletic performance is shaped by the convergence of menstrual-cycle timing, circadian rhythms, fatigue, and ergogenic supplementation; yet no prior study has examined these factors simultaneously in a sport-specific setting. This study investigated the independent and combined effects of acute caffeine ingestion, calendar-based testing window, time of day, and match-induced fatigue on psychophysiological, cognitive, and physical performance in trained female volleyball players. **Methods:** Thirteen elite eumenorrheic female volleyball players (age: 24.23 ± 4.06 years) completed a randomized, double-blind, placebo-controlled crossover protocol comprising 12 sessions corresponding to all combinations of testing window (menstrual, follicular, luteal), supplementation (caffeine 6 mg·kg^−1^ vs. placebo), and time of day (08:00 h vs. 18:00 h). Assessments included the Epworth Sleepiness Scale, Pittsburgh Sleep Quality Index, Spiegel questionnaire, Profile of Mood States, Hooper Index, Stroop task, Countermovement Jump (CMJ), Modified Agility T-Test (MAT), and Reactive Agility Test (RAT), administered before and after a one-hour simulated match. **Results:** Significant main effects of testing window, caffeine, time of day, and fatigue state were observed across all outcome domains (all *p* < 0.05). Caffeine reduced daytime sleepiness (F(1,12) = 23.84, *p* < 0.001, ηp^2^ = 0.665), enhanced vigor (F(1,12) = 114.10, *p* < 0.001, ηp^2^ = 0.905), and improved MAT performance (F(1,12) = 33.27, *p* < 0.001, ηp^2^ = 0.735). The follicular window was associated with superior cognitive, neuromuscular, and mood-related outcomes relative to the menstrual and luteal windows. Exploratory higher-order interactions suggested condition-specific caffeine benefits for MAT, RAT, and CMJ, particularly in afternoon post-fatigue conditions; these patterns require replication in larger samples. **Conclusions:** Acute caffeine ingestion improved several psychophysiological, cognitive, and neuromuscular outcomes in trained female volleyball players, with effects that varied across calendar-based testing windows, time of day, and fatigue state. Individualized supplementation strategies incorporating cycle timing and circadian context remain investigational; prescriptive recommendations require replication in larger, hormonally verified samples before clinical or applied adoption.

## 1. Introduction

Athletic performance in female athletes is governed by a dynamic interaction among hormonal fluctuations across the menstrual cycle, circadian variation in physiological and cognitive readiness, accumulated fatigue from competitive match play, and the modulatory effects of ergogenic supplementation. Despite considerable progress in sports science, the operational complexity of these intersecting determinants has rarely been addressed within a single experimental framework, particularly in team-sport athletes. This gap has practical consequences: training and supplementation recommendations derived predominantly from male cohorts or from studies isolating a single biological variable may inadequately characterize or support female athlete performance [1].

The menstrual cycle produces systematic fluctuations in estradiol and progesterone that extend beyond reproductive function to modulate substrate metabolism, thermoregulation, neuromuscular activation, sleep architecture, and affective state [2]. A systematic review and meta-analysis by McNulty et al. [3] concluded that exercise performance may vary across menstrual-cycle phases, with the early follicular phase associated with relatively less favorable conditions compared to the mid-follicular phase, though the magnitude and direction of effects are inconsistent across individuals and performance tasks. This inter-individual variability underscores the methodological importance of tracking cycle phase with sufficient precision and of resisting overly generalized phase-performance relationships. Beyond neuromuscular outcomes, the menstrual cycle also influences sleep quality, perceived fatigue, mood state, and cognitive function, each of which is a determinant of volleyball performance, a sport requiring repeated explosive actions, rapid attentional switching, and sustained decision-making accuracy under cumulative fatigue [4]. Recent within-individual neuroimaging data indicate that cyclical hormonal fluctuations are associated with brain-wide changes in white matter microstructure and cortical thickness, suggesting that cycle-associated cognitive and performance variability may involve structural and network-level neural reorganization in addition to peripheral physiological modulation [5,6,7].

Superimposed on this hormonal context, circadian biology introduces a parallel source of performance variation. Athletic performance generally follows a diurnal trajectory, with neuromuscular force production, reaction time, and cardiorespiratory efficiency peaking in the mid-to-late afternoon relative to the morning, corresponding to the circadian rise in core body temperature and central arousal [8,9,10]. For team-sport athletes who compete across multiple daily time slots, this circadian architecture has direct operational relevance. Yet whether the magnitude of the morning performance disadvantage varies with menstrual-cycle timing and whether supplementation strategies can simultaneously attenuate both sources of decrement remain largely uncharacterized.

Caffeine is among the most extensively studied ergogenic aids in sport. By competitively antagonizing adenosine A1 and A2A receptors, caffeine attenuates perceived fatigue, increases central nervous system excitability, and enhances neuromuscular recruitment, thereby improving endurance, muscular strength, reaction time, and sport-specific agility across a broad range of athletic tasks [11,12]. An umbrella review of 21 meta-analyses confirmed that acute caffeine ingestion improves exercise performance across multiple domains, with doses of 3–6 mg·kg^−1^ representing the most consistently ergogenic range [11]. Its potential as a chronobiological countermeasure is particularly relevant: caffeine has been shown to attenuate morning performance decrements in both physical and cognitive tasks, effectively compressing the diurnal performance gap [8,13]. However, the growing recognition that caffeine also displaces sleep and increases sleep latency when ingested in the afternoon introduces a performance-recovery trade-off that must be considered in applied settings [14,15].

Despite this evidence base, the interaction between caffeine and menstrual-cycle timing remains poorly characterized. Estradiol has been reported to inhibit cytochrome P450 1A2 (CYP1A2)-mediated hepatic caffeine metabolism, potentially prolonging caffeine half-life in estrogen-elevated calendar windows [16]; without direct pharmacokinetic measurement or hormonal verification, however, this mechanism remains inferential in the present context. If caffeine clearance and receptor sensitivity differ across the menstrual cycle, the ergogenic and adverse effects of a fixed dose may not be uniform across testing windows, with consequences for both performance optimization and side-effect management. Furthermore, match-induced fatigue constitutes an additional modulating factor: central and peripheral fatigue accumulated during competitive play may alter the neurochemical substrate upon which caffeine acts, potentially modifying the magnitude of its benefits on post-match neuromuscular and cognitive outcomes [17,18].

The present study addressed this gap by employing a randomized, double-blind, placebo-controlled crossover design across 12 experimental sessions in elite female volleyball players, assessing a comprehensive battery of psychophysiological, cognitive, and physical performance outcomes before and after a simulated match. The primary aim was to characterize the independent and combined effects of these four factors on performance-relevant outcomes, with the applied objective of informing more individualized and context-sensitive supplementation and preparation strategies for female athletes.

## 2. Methods

### 2.1. Participants

Thirteen elite female volleyball players (age: 24.23 ± 4.06 years; height: 176.25 ± 7.41 cm; body mass: 66.15 ± 12.02 kg; senior competitive experience: 6.77 ± 3.49 years) were recruited from Union Sportive Carthaginoise, competing in the Tunisian Volleyball Premier League. Inclusion criteria were: regular eumenorrheic cycles (25–30 days), no current or recent hormonal contraceptive use (defined as no combined oral, transdermal, injectable, or implantable hormonal contraceptive within three months prior to enrollment); use of non-hormonal contraceptive methods was not an exclusion criterion and was not reported by any participant, no pharmacological treatments or nutritional supplements known to affect endocrine function, sleep, or performance, and no acute or chronic injury. The 25–30-day criterion was selected to minimize within-sample cycle-length variability relative to the broader clinical eumenorrheic range (21–35 days; [1]), thereby reducing calendar-day classification heterogeneity; residual endocrine heterogeneity between participants at the two extremes of this range cannot be excluded, particularly for luteal assessments. Habitual caffeine intake was recorded at enrollment via dietary interview. Participants reported a low habitual caffeine intake (e.g., ≤50 mg/day), classifying them as light caffeine consumers. All participants were instructed to maintain their habitual dietary patterns throughout the study and to abstain from caffeine for 24 h before each session, verified by self-report. Inter-individual differences in habitual caffeine intake represent a potential source of variability in ergogenic responsiveness and sleep-related outcomes [12]. All participants followed a structured training program (five sessions per week, approximately 90 min per session). Chronotype was assessed using the Horne–Östberg Morningness–Eveningness Questionnaire [19]; all participants were classified as intermediate chronotypes, reducing chronobiological bias in time-of-day comparisons.

Sample size was justified a priori using G*Power 3.1 [20]: a minimum of 12 participants was required to detect a moderate within-subject main effect (f = 0.25, α = 0.05, power = 0.80; [21]). This estimation applies to main effects and first-order interactions. Achieving adequate power for three-way or four-way interaction terms in a 3 × 2 × 2 × 2 factorial structure at *n* = 13 is statistically improbable; at this sample size, the risk of Type I error inflation and partial eta squared overestimation for interaction terms is substantial. Accordingly, all three-way and four-way interaction effects are designated a priori as exploratory and must not be interpreted as confirmatory evidence. The protocol was approved by the University of Jendouba Ethics Committee (approval number: C-0018/2024; 18 November 2024) and conducted in accordance with the Declaration of Helsinki. Written informed consent was obtained from all participants and coaching staff.

### 2.2. Study Design

**Figure 1:**
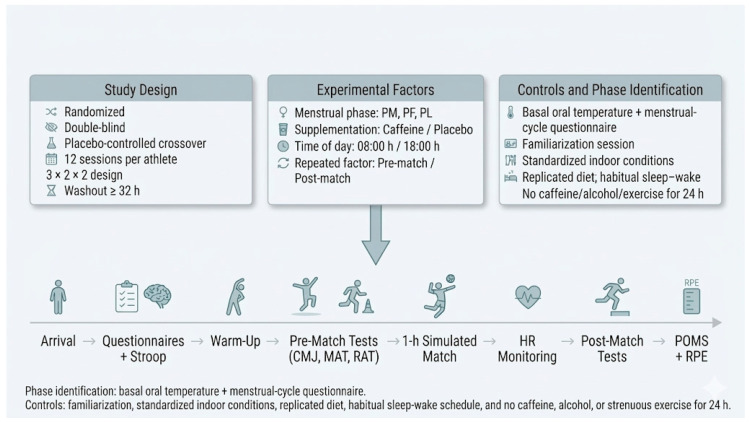
Experimental protocol evaluating the influence of time of day and menstrual-cycle phase on the ergogenic effects of caffeine. Phase identification: basal oral temperature + menstrual-cycle questionnaire. PM: menstrual phase (days 2–4); PF: follicular phase (days 7–9); PL: luteal phase (days 19–21). Replicated diet: participants recorded and reproduced pre-testing meal composition and timing for 24 h preceding each session; habitual sleep–wake: consistent sleep onset (±30 min) maintained for 48 h before each session.

A randomized, double-blind, placebo-controlled, repeated-measures crossover design was employed. Each participant completed 12 experimental sessions across all combinations of calendar-based testing window (menstrual, mid-follicular, mid-luteal), supplementation condition (caffeine vs. placebo), and time of day (08:00 h vs. 18:00 h), yielding a 3 × 2 × 2 within-subjects design. Fatigue state (pre- vs. post-match) served as an additional repeated factor for variables assessed before and after the simulated match (Figure 1). A minimum 32 h inter-session washout was imposed to minimize caffeine carryover, substantially exceeding the compound’s acute ergogenic window. The 32 h minimum washout substantially exceeds caffeine’s acute ergogenic window (estimated 4–6 h; [12]) and was judged sufficient for neuromuscular recovery from a one-hour simulated match in conditioned athletes maintaining their regular training schedule. No systematic pre-match performance drift across sessions was identified in the descriptive data. Complete neuromuscular recovery within 32 h cannot be guaranteed and represents a minor limitation. Morning (08:00 h) and afternoon (18:00 h) testing sessions were conducted on separate days. Within each testing window, each participant completed both time-of-day conditions for each supplementation condition on non-consecutive days; session order (morning-first vs. afternoon-first) within each window was counterbalanced across participants using a balanced block randomization scheme. Participants maintained their habitual sleep–wake schedule throughout the study, confirmed via daily self-report log; sleep onset and wake times were required to remain within ±30 min of each participant’s individually recorded habitual schedule for the 48 h preceding each session. Across-session condition order was assigned using a counterbalanced Latin-square-based allocation scheme, ensuring each of the 12 conditions appeared in each serial position with approximately equal frequency across participants, minimizing systematic order effects. Residual carryover from accumulated competitive load, seasonal fitness variation, or progressive learning across multiple cycles cannot be fully excluded. Because four sessions per testing window at minimum 32 h intervals require approximately 96 h, and each predefined window spans approximately three days, sessions were distributed across at least two consecutive menstrual cycles per participant; longitudinal fitness drift was considered negligible given the stable competitive training load maintained throughout data collection. One week before data collection, all participants completed a single familiarization session. We acknowledge that a single session may not fully eliminate performance adaptation in high learning-curve tasks, such as the reactive agility test (RAT) and computerized Stroop paradigm. Additionally, no intraclass correlation coefficient (ICC) or coefficient of variation (CV) data were computed to verify pre-experimental measurement stability. The absence of reliability verification represents a constraint on the interpretability of performance data, particularly from early experimental sessions. Participants maintained their regular competitive training schedule (five sessions per week, approximately 90 min per session) throughout data collection and were instructed to refrain from strenuous exercise and any ergogenic supplementation for 24 h before each session. Systematic objective training load monitoring across the full multi-cycle period was not performed; fluctuations in competitive demands between cycles represent an uncontrolled variable and a minor limitation.

To minimize nutritional confounds on caffeine absorption and physical performance, participants recorded their pre-session dietary intake (macronutrient composition and meal timing) on a structured 24 h dietary log provided by the research team and were instructed to replicate this across all sessions. The final pre-session meal was consumed no later than 2 h before testing; caffeine-containing foods, beverages, and supplements were prohibited for 24 h before each session.

### 2.3. Calendar-Based Testing Window Identification

Basal oral temperature was recorded at home immediately upon waking, before rising or consuming food or fluid, using a calibrated digital basal thermometer (0.01 °C resolution); participants logged readings daily in a standardized paper diary from cycle day 1. Testing windows were identified using daily basal oral temperature monitoring combined with a validated menstrual-cycle questionnaire recording cycle length, menstruation onset, bleeding characteristics, and symptom profile [22]. Eumenorrheic status was verified prospectively across two full cycles before the experimental period. A sustained post-ovulatory temperature elevation of ≥0.2 °C maintained for ≥3 consecutive days confirmed luteal phase onset; the menstrual onset was defined as the first day of frank bleeding; the mid-follicular window was identified by day count from onset in the absence of an ovulatory temperature shift. Convergence of temperature data and questionnaire responses classified sessions within predefined menstrual (days 2–4), mid-follicular (days 7–9), or mid-luteal (days 19–21) windows. Participants were monitored for a minimum of two complete menstrual cycles before experimental sessions commenced, beginning at cycle day 1 (first day of frank bleeding). Daily temperature and symptom data were recorded in standardized paper logs supplemented by a validated cycle-tracking application. No participant entered the experimental phase until cycle regularity across two consecutive cycles had been confirmed. For participants with 25-day cycles, days 19–21 correspond to approximately 5–7 days post-estimated ovulation; for those with 30-day cycles, the same window corresponds to approximately 8–10 days post-ovulation. This variability introduces uncertainty regarding progesterone peak status in participants with shorter cycles and constitutes a recognized endocrine heterogeneity constraint on luteal-window-specific inferences [1]. The mid-follicular window (days 7–9) was selected to represent a stable, rising-estradiol, low-progesterone period while avoiding the periovulatory LH surge window (typically days 10–14), which requires LH monitoring for reliable classification. Although estradiol peaks during the late follicular/periovulatory phase, identifying the periovulatory period solely via calendar methods without urinary luteinizing hormone (LH) testing presents substantial classification challenges; therefore, we selected a mid-follicular window to minimize calendar-based estimation errors [1,3]. No urinary LH testing or serum hormonal assays were performed. These windows, therefore, represent calendar-based classifications and must not be equated with biochemically verified endocrine phases. Basal oral temperature monitoring is inherently retrospective and has limited precision for ovulation identification, being considerably less accurate than urinary LH testing or serum hormonal assays [1]. This constitutes a recognized methodological limitation constraining all testing window-related inferences. Menstrual symptom severity (dysmenorrhea, cramping, bloating, and headache) was not formally quantified using a validated symptom scale. Participants reporting acute illness or severe menstrual symptoms precluding normal training participation were rescheduled. Residual variation in symptom burden across menstrual-window sessions represents a potential confound that may have contributed to the elevated Hooper Index and mood disturbance scores observed in this window.

Urinary LH surge testing and serum hormonal assays were not incorporated owing to logistical constraints in the competitive training environment and the practical difficulty of standardizing LH test timing relative to the variable ovulation window in an athletic cohort with demanding schedules. The calendar-basal-temperature approach was selected as a validated, minimally invasive, field-applicable method consistent with prior applied research contexts [1,3]. All cycle-related inferences are strictly bounded to testing window comparisons rather than confirmed endocrine states.

### 2.4. Caffeine and Placebo Administration

Caffeine (CAF) and placebo (PLC) capsules were visually identical, odorless, and tasteless; allocation was concealed from participants and investigators throughout data collection. In CAF sessions, participants ingested 6 mg·kg^−1^ anhydrous caffeine (PRO PLUS™, UK; 100% purity); in PLC sessions, an identical capsule containing microcrystalline cellulose (Guinama 6™, Spain) was administered. All capsules were taken orally with 100 mL of water 60 min before testing, corresponding to peak plasma caffeine concentration [23,24]. Blinding effectiveness was not assessed with a post-session condition-guessing questionnaire. Given that 6 mg·kg^−1^ caffeine may produce discernible somatic cues, including elevated arousal and tachycardia, condition identification by participants cannot be excluded; expectancy effects may consequently have influenced subjective perceptual outcomes, including the rating of perceived exertion (RPE), Profile of Mood States (POMS) dimensions, and the Hooper Index, and these measures must be interpreted with this caveat.

### 2.5. Measurements

Upon arrival, participants completed psychometric and sleep assessments: the Profile of Mood States (POMS; [25]), Hooper Index (HI; [26], a four-item composite questionnaire assessing perceived sleep quality, fatigue, stress, and muscle soreness; scored 4–28 with higher values indicating greater psychophysiological strain), Pittsburgh Sleep Quality Index (PSQI; [27]), Epworth Sleepiness Scale (ESS; [28]), and Spiegel Sleep Questionnaire [29]. Objective sleep monitoring via actigraphy or polysomnography was not performed; sleep data are based exclusively on validated self-report instruments (PSQI and Spiegel questionnaire). Executive and attentional function were assessed using a computerized Stroop task (color-naming, word-reading, and interference conditions; [30,31]), recording total response time and error count. Following a standardized 10 min warm-up, physical performance was assessed in fixed order: Countermovement Jump (CMJ) height via the Optojump Next infrared system (Microgate, Bolzano, Italy; [32]), change-of-direction speed via the Modified Agility T-Test (MAT) [33,34] using Brower dual timing gates (Brower Timing Systems, Draper, UT, USA, precision: 0.01 s), and reactive agility via the RAT [35] using a 7 × 5 LED Smart Indicator Matrix (Fusion Sport, Brisbane, Australia). During the subsequent one-hour simulated match. The simulated match was structured to replicate competitive volleyball demands, comprising 15 min of standardized technical sequences (serving, spiking, blocking, and defensive actions) followed by 45 min of game-play sets (e.g., 6 vs. 6 format) conducted under coaching supervision to maintain continuous high-intensity competitive efforts. Match structure and workload were held constant across all sessions to equate the fatigue stimulus; continuous heart rate monitoring confirmed that exercise intensity was consistent with competitive match play. Estimated heart rate was recorded continuously using the Polar Team Sport System (Polar Electro, Kempele, Finland), with transmitter belts positioned on the sternum. Recording commenced at match onset and ended at the final whistle; the %HRavg was computed across this match epoch exclusively, with the pre-match warm-up period excluded from the analysis [36]. Post-match, CMJ, MAT, and RAT were repeated, and session-end perceived exertion was quantified using the Borg RPE scale (range 6–20, where 6 = no exertion and 20 = maximal exertion; [37]). Adverse effects were monitored post-session and at 24 h follow-up immediately using a structured 10-item Side-Effect Inventory rated on a 3-point scale (0 = absent, 1 = mild, 2 = moderate; [38,39]). The Polar Team Sport System provides processed beat-to-beat HR estimates derived from proprietary algorithms rather than raw ECG waveforms; independent signal-quality verification from exported data is not possible.

### 2.6. Statistical Analysis

**Figure 2:**
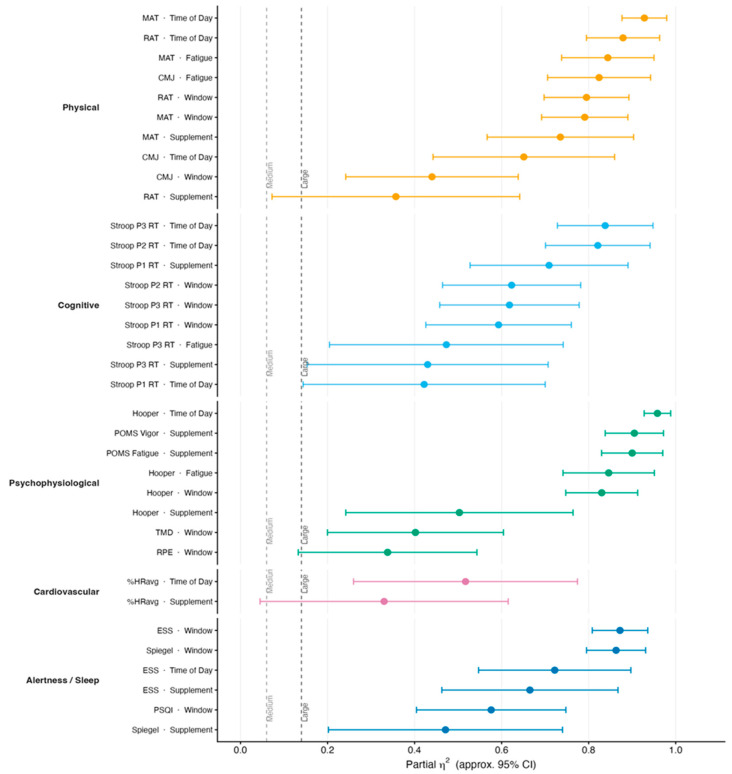
Summary of effect sizes (ηp^2^) for all significant ANOVA main effects across outcome domains. Forest plot displays ηp^2^ estimates with 95% confidence intervals derived from F-statistic transformation. Outcomes are grouped by domain (physical performance, psychophysiological, sleep, cognitive, cardiovascular). Vertical reference lines indicate Cohen’s conventional thresholds for medium (0.06) and large (0.14) effects. Within-athlete repeated-measures designs typically yield inflated ηp^2^ due to reduced error variance; this plot is intended as a descriptive summary rather than a basis for cross-domain comparison.

All analyses were performed using STATISTICA version 13.3 (StatSoft, France) and R version 4.3.2 [40]. Figure production and effect-size visualization employed the following R packages: ggplot2 (v3.5.0), ggdist (v3.3.1), patchwork (v1.2.0), dplyr (v1.1.4), forcats (v1.0.0), and scales (v1.3.0). CMJ, MAT, and RAT were the primary outcomes; psychophysiological, sleep, perceptual, cardiovascular, and cognitive variables were secondary outcomes. For variables assessed pre- and post-match, a four-way repeated-measures ANOVA examined the main effects and interactions of calendar-based testing window (menstrual, follicular, luteal), supplementation (CAF vs. PLC), time of day (morning vs. afternoon), and fatigue state. A three-way ANOVA (window × supplementation × time of day) was applied to ESS, %HRavg, and RPE; a two-way ANOVA (window × supplementation) was applied to PSQI and Spiegel scores. Greenhouse–Geisser correction was applied when sphericity was violated. Significant effects were followed by Bonferroni-adjusted post hoc comparisons. Effect sizes are reported as partial eta squared (ηp^2^; small: 0.01; medium: 0.06; large: 0.14); approximate 95% confidence intervals for ηp^2^ derived from F-statistic transformation are presented in Figure 2, interpreted contextually given that repeated-measures designs in elite athlete samples reduce within-subject error variance and may inflate ηp^2^ estimates relative to between-subjects benchmarks. Pairwise effect magnitudes are reported as Cohen’s d. Readers are encouraged to prioritize Cohen’s dz as the primary practical significance index alongside *p*-values; the large ηp^2^ values characteristic of repeated-measures athlete designs should be interpreted conservatively given systematic within-subject error suppression [1]. Exact *p*-values are reported throughout; values below 0.001 are stated as *p* < 0.001. Side-effect frequencies were compared using McNemar exact tests with Bonferroni correction. All three-way and four-way interaction effects are designated exploratory a priori, characterizing condition-specific patterns for hypothesis generation rather than confirmatory inference [41]. Missing data were not imputed; no observations were excluded post-randomization. Statistical significance was set at *p* < 0.05.

## 3. Results

### 3.1. Primary Physical Performance Outcomes

**Figure 3:**
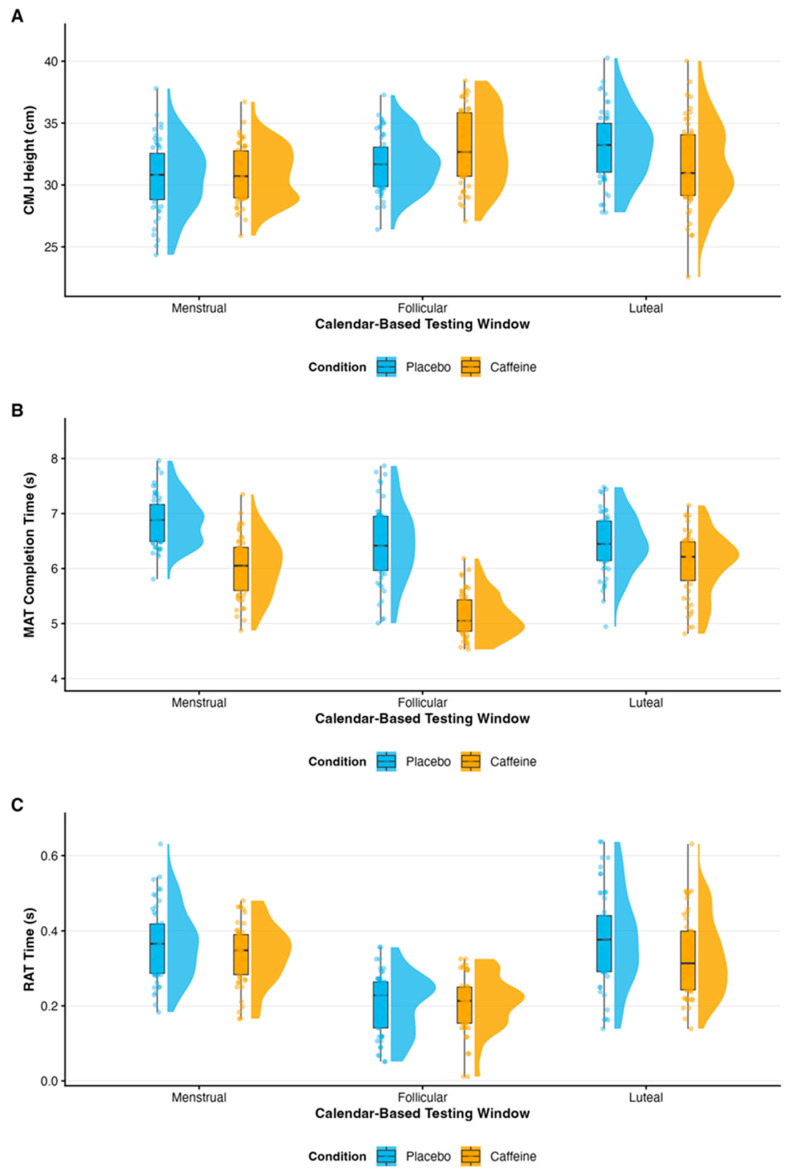
Primary physical performance outcomes by supplementation condition and calendar-based testing window. Raincloud plots display the distribution of (**A**) Countermovement Jump height (cm), (**B**) Modified Agility T-Test completion time (s), and (**C**) Reactive Agility Test time (s) across caffeine (CAF) and placebo (PLC) conditions, stratified by testing window (menstrual, follicular, luteal). Each panel combines a half-violin density estimate, individual data points (jittered), and a boxplot. Error bars within boxplots represent the median and interquartile range. Asterisks denote Bonferroni-corrected significant caffeine–placebo differences within a window where visually distinguishable. Colorblind-safe palette (Okabe-Ito).

Countermovement Jump: Significant main effects were detected for calendar-based testing window (F(2,24) = 9.43, *p* < 0.001, ηp^2^ = 0.440), time of day (F(1,12) = 22.42, *p* < 0.001, ηp^2^ = 0.651), and fatigue state (F(1,12) = 56.17, *p* < 0.001, ηp^2^ = 0.824). The caffeine main effect did not reach significance (F(1,12) = 4.17, *p* = 0.063, ηp^2^ = 0.258). CMJ was lower in the menstrual window than in both the follicular (*p* = 0.003, dz = 1.23) and luteal windows (*p* = 0.003, dz = 1.18); the follicular and luteal windows did not differ. Match play induced a substantial decline across all conditions (*p* < 0.001, dz = 2.08). A significant Testing Window × Time of Day interaction (F(2,24) = 8.88, *p* = 0.001, ηp^2^ = 0.426) indicated that the afternoon advantage in CMJ was present during the follicular and luteal windows but absent during the menstrual window. Three exploratory terms were also identified: Caffeine × Time of Day (F(1,12) = 7.38, *p* = 0.019, ηp^2^ = 0.381), Caffeine × Fatigue (F(1,12) = 8.69, *p* = 0.012, ηp^2^ = 0.420), and the three-way Caffeine × Time of Day × Fatigue interaction (F(1,12) = 14.30, *p* = 0.003, ηp^2^ = 0.544). Caffeine improved CMJ in the afternoon (*p* = 0.032, dz = 0.78) and post-fatigue (*p* = 0.028, dz = 0.80) conditions, but not in the morning or pre-fatigue. The largest exploratory cell-specific difference was observed in the afternoon post-fatigue condition (*p* = 0.007, dz = 1.12); this comparison requires replication in larger samples (Figure 3).

Modified Agility T-Test: All four main effects were significant: testing window (F(2,24) = 45.19, *p* < 0.001, ηp^2^ = 0.791), caffeine (F(1,12) = 33.27, *p* < 0.001, ηp^2^ = 0.735), time of day (F(1,12) = 154.55, *p* < 0.001, ηp^2^ = 0.928), and fatigue state (F(1,12) = 64.80, *p* < 0.001, ηp^2^ = 0.844). Caffeine reduced MAT completion time overall. A significant Time of Day × Fatigue interaction (F(1,12) = 9.61, *p* = 0.009, ηp^2^ = 0.444) indicated that the fatigue-related MAT decrement was more pronounced in the afternoon. The following terms are designated exploratory: Testing Window × Caffeine (F(2,24) = 26.45, *p* < 0.001, ηp^2^ = 0.688), Caffeine × Time of Day (F(1,12) = 44.31, *p* < 0.001, ηp^2^ = 0.787), Testing Window × Fatigue (F(2,24) = 11.96, *p* < 0.001, ηp^2^ = 0.499), Testing Window × Caffeine × Fatigue (F(2,24) = 11.55, *p* < 0.001, ηp^2^ = 0.490), and the four-way Testing Window × Caffeine × Time of Day × Fatigue interaction (F(2,24) = 5.88, *p* = 0.008, ηp^2^ = 0.329). Exploratory post hoc patterns indicated that caffeine improved MAT across all four conditions during the follicular window (dz range: 1.52–2.09) and was restricted to afternoon sessions in the menstrual window (pre-fatigue: *p* < 0.001, dz = 1.87; post-fatigue: *p* < 0.001, dz = 2.04) and to afternoon sessions in the luteal window (pre-fatigue: *p* = 0.022, dz = 1.10; post-fatigue: *p* = 0.011, dz = 1.22), with no significant morning effects in either the menstrual or luteal windows.

Reactive Agility Test: Significant main effects were observed for testing window (F(2,24) = 46.47, *p* < 0.001, ηp^2^ = 0.795), caffeine (F(1,12) = 6.65, *p* = 0.024, ηp^2^ = 0.357), and time of day (F(1,12) = 86.84, *p* < 0.001, ηp^2^ = 0.879). Fatigue had no significant main effect (F(1,12) = 0.01, *p* = 0.908, ηp^2^ = 0.001). Caffeine reduced RAT time overall (*p* = 0.024, dz = 0.72). Exploratory interactions included: Testing Window × Caffeine (F(2,24) = 4.23, *p* = 0.027, ηp^2^ = 0.261), Caffeine × Fatigue (F(1,12) = 14.64, *p* = 0.002, ηp^2^ = 0.550), Caffeine × Time of Day (F(1,12) = 7.04, *p* = 0.021, ηp^2^ = 0.370), and the three-way Caffeine × Time of Day × Fatigue (F(1,12) = 8.65, *p* = 0.012, ηp^2^ = 0.419); the four-way interaction was not significant (F(2,24) = 1.49, *p* = 0.246, ηp^2^ = 0.110). Following Bonferroni correction across 12 testing window × time × fatigue comparisons, significant caffeine effects were retained in the menstrual window morning pre-fatigue (*p* = 0.008, dz = 1.27), menstrual window afternoon pre-fatigue (*p* = 0.010, dz = 1.23), luteal window morning pre-fatigue (*p* < 0.001, dz = 1.87), and luteal window afternoon pre-fatigue (*p* = 0.010, dz = 1.22). No significant caffeine effects were observed in post-fatigue conditions or in the follicular window after correction. Complete descriptive statistics are presented in Table 1.

### 3.2. Psychophysiological and Perceptual Outcomes

Profile of Mood States: Caffeine significantly reduced POMS fatigue (F(1,12) = 107.46, *p* < 0.001, ηp^2^ = 0.900) and increased vigor (F(1,12) = 114.10, *p* < 0.001, ηp^2^ = 0.905). The Supplementation × Fatigue interaction for POMS fatigue was non-significant (F(1,12) = 0.41, *p* = 0.534, ηp^2^ = 0.033); given the sample size, this result should not be interpreted as confirming the absence of interaction, as a Type II error cannot be excluded. For vigor, a significant Supplementation × Fatigue interaction (F(1,12) = 7.03, *p* = 0.021, ηp^2^ = 0.370) indicated that caffeine attenuated the post-match vigor decline observed under placebo (dz = 1.65). A significant Supplementation × Fatigue interaction for confusion (F(1,12) = 17.64, *p* = 0.001, ηp^2^ = 0.595) showed that post-match confusion increased under placebo (*p* < 0.01) but not under caffeine. Anxiety and anger exhibited significant fatigue-state main effects (both *p* < 0.05) without significant supplementation effects or interactions; depression remained unchanged. Total mood disturbance (TMD) showed a significant testing window effect (F(2,24) = 8.07, *p* = 0.002, ηp^2^ = 0.402; Greenhouse–Geisser-corrected *p* = 0.014), with lower scores in the follicular window than in both the menstrual (*p* < 0.001) and luteal windows (*p* = 0.004). Full POMS data are presented in Table 2.

Hooper Index: All four main effects were significant: testing window (F(2,24) = 58.75, *p* < 0.001, ηp^2^ = 0.830), supplementation (F(1,12) = 12.12, *p* = 0.005, ηp^2^ = 0.503), time of day (F(1,12) = 270.52, *p* < 0.001, ηp^2^ = 0.958), and fatigue state (F(1,12) = 65.84, *p* < 0.001, ηp^2^ = 0.846). The only significant interaction was Time of Day × Fatigue (F(1,12) = 5.18, *p* = 0.042, ηp^2^ = 0.302). Hooper scores were highest in the menstrual window (14.46 ± 1.49), lower in the luteal window (13.71 ± 0.90), and lowest in the follicular window (10.52 ± 0.89; both *p* < 0.001). Placebo scores exceeded caffeine scores (13.12 ± 0.99 vs. 12.67 ± 0.62; *p* = 0.005, dz = 0.97). Morning values exceeded afternoon values (13.76 ± 0.86 vs. 12.04 ± 0.76; *p* < 0.001, dz = 4.56). Simple effects confirmed that fatigue-related Hooper decrements were significant in both morning (14.24 ± 0.86 vs. 13.27 ± 0.92; *p* < 0.001, dz = 2.11) and afternoon sessions (12.62 ± 0.74 vs. 11.46 ± 0.87; *p* < 0.001, dz = 2.20). Hooper’s data are presented in Table 3.

Rating of Perceived Exertion: A significant testing window main effect was observed (F(2,24) = 6.13, *p* = 0.007, ηp^2^ = 0.338), with no significant caffeine (F(1,12) = 1.89, *p* = 0.193) or time of day (F(1,12) = 4.15, *p* = 0.063) main effects. A significant Testing Window × Caffeine interaction (F(2,24) = 4.89, *p* = 0.017, ηp^2^ = 0.290) indicated that caffeine-related RPE reductions were window-specific. RPE and cardiovascular load data are presented in Table 4.

### 3.3. Sleep-Related and Alertness Outcomes

Epworth Sleepiness Scale: Testing window (F(2,24) = 81.54, *p* < 0.001, ηp^2^ = 0.872), supplementation (F(1,12) = 23.84, *p* < 0.001, ηp^2^ = 0.665), and time of day (F(1,12) = 31.23, *p* < 0.001, ηp^2^ = 0.722) each exerted independent significant effects on ESS scores. All two-way and three-way interactions were non-significant (all *p* > 0.47). ESS was highest in the menstrual window (7.87 ± 1.01), intermediate in the luteal window (6.88 ± 0.96), and lowest in the follicular window (5.50 ± 0.80; all pairwise comparisons *p* < 0.002). Caffeine reduced ESS relative to placebo (6.62 ± 1.28 vs. 6.88 ± 1.40; *p* < 0.001, dz = 1.35), and morning values exceeded afternoon values (7.01 ± 1.26 vs. 6.49 ± 1.37; *p* < 0.001, dz = 1.55).

Pittsburgh Sleep Quality Index: A significant testing window effect (F(2,24) = 16.32, *p* < 0.001, ηp^2^ = 0.576) indicated worse sleep quality in both the menstrual (6.23 ± 0.86) and luteal (5.96 ± 1.34) windows relative to the follicular window (4.04 ± 0.72; *p* < 0.001, dz = 2.01 and *p* = 0.001, dz = 1.30, respectively); the menstrual and luteal windows did not differ (*p* = 0.610). Caffeine did not significantly affect PSQI scores (F(1,12) = 0.71, *p* = 0.416, ηp^2^ = 0.056), and the Testing Window × Supplementation interaction was non-significant (F(2,24) = 1.00, *p* = 0.383, ηp^2^ = 0.077).

Spiegel Sleep Questionnaire: Testing window (F(2,24) = 75.34, *p* < 0.001, ηp^2^ = 0.863) and supplementation (F(1,12) = 10.69, *p* = 0.007, ηp^2^ = 0.471) exerted significant effects; their interaction was non-significant (F(2,24) = 0.05, *p* = 0.955, ηp^2^ = 0.004). Spiegel scores were substantially higher in the luteal window (24.35 ± 1.02) than in both the follicular (20.73 ± 1.04; *p* < 0.001, dz = 3.12) and menstrual windows (20.23 ± 1.14; *p* < 0.001, dz = 3.73); follicular and menstrual windows did not differ (*p* = 0.291). Caffeine yielded marginally higher Spiegel scores than placebo (21.95 ± 2.09 vs. 21.59 ± 2.17; *p* = 0.007, dz = 0.91). Sleep-related outcomes are presented in Table 5.

### 3.4. Cognitive Performance

Reaction Time: For Stroop Plate 1 (color naming), significant main effects of testing window (F(2,24) = 17.49, *p* < 0.001, ηp^2^ = 0.593), caffeine (F(1,12) = 29.28, *p* < 0.001, ηp^2^ = 0.709), and time of day (F(1,12) = 8.77, *p* = 0.012, ηp^2^ = 0.422) were observed. A significant Testing Window × Time of Day interaction (F(2,24) = 15.47, *p* < 0.001, ηp^2^ = 0.563) indicated that the afternoon advantage was significant only during the menstrual and luteal windows. Caffeine reduced Plate 1 reaction time overall (*p* < 0.001, dz = 1.50). Follicular window reaction times were shorter than both menstrual (*p* < 0.001, dz = 1.56) and luteal windows (*p* < 0.001, dz = 1.65). For Plate 2 (word reading), significant testing window (F(2,24) = 19.79, *p* < 0.001, ηp^2^ = 0.623) and time of day effects (F(1,12) = 54.88, *p* < 0.001, ηp^2^ = 0.821) were observed; caffeine approached but did not reach significance (F(1,12) = 4.52, *p* = 0.055, ηp^2^ = 0.274). For Plate 3 (interference condition), significant main effects were found for testing window (F(2,24) = 19.37, *p* < 0.001, ηp^2^ = 0.618), caffeine (F(1,12) = 9.04, *p* = 0.011, ηp^2^ = 0.430), time of day (F(1,12) = 62.10, *p* < 0.001, ηp^2^ = 0.838), and fatigue (F(1,12) = 10.76, *p* = 0.007, ηp^2^ = 0.473). A significant exploratory Caffeine × Fatigue interaction (F(1,12) = 21.31, *p* < 0.001, ηp^2^ = 0.640) indicated that caffeine attenuated the fatigue-related increase in interference reaction time observed under placebo (*p* < 0.001, dz = 1.21), whereas no significant increase was observed under caffeine.

Errors: Across Plates 1 and 2, significant main effects of testing window, caffeine, and time of day were consistently observed (all *p* < 0.007; ηp^2^ range: 0.369–0.936). Errors were lower in the follicular window than in both other windows (all *p* < 0.031), lower in the afternoon (Plate 2 afternoon vs. morning: dz = 3.67), and reduced under caffeine (Plate 1: *p* = 0.007, dz = 0.89; Plate 2: *p* = 0.005, dz = 0.94). Plate 3 errors showed significant testing window (F(2,24) = 15.41, *p* < 0.001, ηp^2^ = 0.562) and time-of-day effects (F(1,12) = 118.86, *p* < 0.001, ηp^2^ = 0.908), without significant caffeine or fatigue effects. Full Stroop data are presented in Table 6.

### 3.5. Cardiovascular Load

Caffeine significantly reduced %HRavg overall (F(1,12) = 5.92, *p* = 0.032, ηp^2^ = 0.330; caffeine: 55.20 ± 4.84% vs. placebo: 58.49 ± 2.88%; dz = 0.67). Time of day yielded a significant main effect (F(1,12) = 12.86, *p* = 0.004, ηp^2^ = 0.517), with higher values in the morning (58.20 ± 2.82% vs. 55.49 ± 3.95%; dz = 0.99). Note that these cardiovascular load values represent estimates derived from the wearable monitoring device. The main effect of the testing window did not reach significance (F(2,24) = 3.17, *p* = 0.060, ηp^2^ = 0.209). A significant Testing Window × Caffeine interaction (F(2,24) = 5.88, *p* = 0.008, ηp^2^ = 0.329) indicated window-specific caffeine effects: caffeine significantly reduced %HRavg in the follicular window afternoon (49.11 ± 9.21% vs. 56.84 ± 7.09%; *p* = 0.014, dz = 0.80) and in both morning and afternoon sessions of the luteal window (morning: *p* = 0.033, dz = 0.67; afternoon: *p* = 0.036, dz = 0.66), whereas no significant caffeine effect was detected in the menstrual window (morning: *p* = 0.138; afternoon: *p* = 0.434). Cardiovascular data are presented in Table 4.

### 3.6. Adverse Effects

Overall, caffeine was well tolerated; reported symptoms were predominantly mild and transient. McNemar exact tests indicated that caffeine was associated with a higher frequency of self-reported performance enhancement (*p* < 0.001) and insomnia complaints (*p* = 0.002) immediately post-session. The higher frequency of increased-activity reports under caffeine (*p* = 0.021) did not survive Bonferroni correction. At the 24 h follow-up, only increased-activity reports remained significantly more frequent following caffeine (*p* < 0.001). Exploratory window-specific patterns suggested heightened activity perception and performance enhancement during the menstrual window, and greater insomnia incidence during the follicular window; these subgroup observations are descriptive, given low cell frequencies. Afternoon caffeine sessions were associated with a numerically higher incidence of insomnia complaints relative to morning sessions, consistent with pharmacokinetic evidence on caffeine-related sleep-onset delay [14,15]; formal between-time-of-day adverse-effect comparisons were not conducted owing to insufficient cell frequencies.

## 4. Discussion

The present study is, to our knowledge, the first to simultaneously examine the independent and combined effects of acute caffeine ingestion, calendar-based testing window, time of day, and match-induced fatigue on a comprehensive battery of psychophysiological, cognitive, and physical performance outcomes in elite female volleyball players. The principal findings indicate that all four factors independently influenced at least one outcome domain, and that caffeine-related benefits were condition-specific rather than globally uniform. These observations extend the existing ergogenic literature by situating caffeine’s effects within an integrative chronobiological and menstrual-cycle context that more closely reflects the operational demands of competitive female sport.

Performance and well-being outcomes were consistently more favorable during the follicular testing window relative to the menstrual and luteal windows. The follicular window was associated with lower ESS scores (daytime sleepiness; 5.50 ± 0.80 vs. 7.87 ± 1.01 during the menstrual window; *p* < 0.001, dz = 3.29), lower PSQI scores (retrospective sleep quality; 4.04 ± 0.72 vs. 6.23 ± 0.86; *p* < 0.001, dz = 2.01), shorter Stroop reaction times across all three plates, and superior CMJ, MAT, and RAT performance. These patterns are broadly consistent with systematic evidence associating the mid-follicular calendar window with more favorable physiological and psychological states [2,3]. However, because no hormonal assays were conducted in the present study, these differences must be attributed strictly to calendar-based window effects, and endocrine mechanistic interpretations are not warranted. This constraint aligns with Elliott-Sale et al. [1], who emphasized that calendar-based classification introduces substantial classification uncertainty and should not be conflated with biochemically confirmed endocrine states. The menstrual window was also associated with the highest Hooper Index scores (composite psychophysiological readiness; 14.46 ± 1.49) and the greatest total mood disturbance (F(2,24) = 8.07, *p* = 0.002, ηp^2^ = 0.402), consistent with prior evidence that perceptual and affective strain is elevated during menstruation in athletic populations [4,39].

Caffeine ingestion significantly reduced daytime sleepiness (F(1,12) = 23.84, *p* < 0.001, ηp^2^ = 0.665), enhanced POMS vigor (F(1,12) = 114.10, *p* < 0.001, ηp^2^ = 0.905), and reduced perceived fatigue (F(1,12) = 107.46, *p* < 0.001, ηp^2^ = 0.900). These findings are consistent with caffeine’s established mechanism of adenosine-receptor antagonism, which attenuates perceived fatigue and elevates central nervous system activation [12,18]. The significant Supplement × Fatigue interaction for vigor (F(1,12) = 7.03, *p* = 0.021, ηp^2^ = 0.370) indicated that caffeine attenuated the post-match decline in perceived readiness, a finding with practical relevance for volleyball, where sustained attentional engagement and explosive output are required throughout the full match. Comparable attenuation of fatigue-related affective decrements has been reported in female handball players following caffeine ingestion during Ramadan fasting [39] and in mixed athletic samples [42,43]. The simulated match replicated the physical and technical demands of competitive volleyball; however, it lacked the full psychological load of official competition (score pressure, opponent interaction, official adjudication) and was conducted under standardized controlled conditions. Ecological validity relative to official match conditions was therefore partial. Caffeine did not significantly alter PSQI scores (*p* = 0.416), suggesting that acute pre-session caffeine at the doses and timing used does not systematically impair retrospective sleep quality, although the higher frequency of insomnia complaints under caffeine immediately post-session warrants caution, particularly when sessions occur in the afternoon, consistent with pharmacokinetic evidence that caffeine ingested six or more hours before habitual sleep onset can still measurably reduce sleep duration and quality [14,15]. The apparent divergence between the PSQI and Spiegel trajectories across testing windows warrants brief methodological clarification: the PSQI indexes retrospective global sleep quality across the preceding month, whereas the Spiegel questionnaire captures subjective sleep quality for the single night immediately preceding each session. The luteal window’s combination of the highest PSQI scores and the highest Spiegel scores is therefore not paradoxical; it reflects that athletes may report globally impaired sleep across the luteal phase as a whole while the specific night assessed by the Spiegel questionnaire was, on average, adequate, possibly because testing sessions were not systematically scheduled at the nadir of nocturnal symptom severity within that window. Because POMS subscales (vigor, fatigue, confusion), the Hooper Index, and RPE are perceptual measures, expectancy effects associated with the distinctive somatic profile of 6 mg·kg^−1^ caffeine (elevated arousal, tachycardia) cannot be excluded, given that blinding effectiveness was not formally verified via a condition-guessing questionnaire. These measures should be interpreted with this caveat.

Caffeine significantly reduced Stroop Plate 1 reaction time (F(1,12) = 29.28, *p* < 0.001, ηp^2^ = 0.709) and Plate 3 interference reaction time (F(1,12) = 9.04, *p* = 0.011, ηp^2^ = 0.430), with an exploratory Supplement × Fatigue interaction for Plate 3 (F(1,12) = 21.31, *p* < 0.001, ηp^2^ = 0.640) indicating that caffeine prevented the fatigue-related increase in interference processing time observed under placebo. These results corroborate evidence that caffeine preserves executive function under conditions of accumulated neural fatigue [11,18], and are particularly relevant given that volleyball performance depends on rapid decision-making under fatigue. Afternoon sessions produced systematically faster reaction times and fewer errors across all Stroop plates relative to morning sessions, consistent with circadian models of cognitive arousal that describe a diurnal rise in alertness and processing speed peaking in the mid-to-late afternoon [8]. The Phase × Time of Day interaction for Plate 1 (F(2,24) = 15.47, *p* < 0.001, ηp^2^ = 0.563) indicated that the afternoon cognitive advantage was significant specifically during the menstrual and luteal windows, suggesting that circadian-driven improvements may partially compensate for window-related cognitive decrements.

Caffeine exerted a significant main effect on MAT performance (F(1,12) = 33.27, *p* < 0.001, ηp^2^ = 0.735) and RAT performance (F(1,12) = 6.65, *p* = 0.024, ηp^2^ = 0.357), but not on CMJ (F(1,12) = 4.17, *p* = 0.063, ηp^2^ = 0.258). The absence of a significant caffeine main effect on CMJ contrasts with some prior evidence of caffeine-related improvements in jump height [17], though the significant exploratory Caffeine × Time of Day × Fatigue interaction (F(1,12) = 14.30, *p* = 0.003, ηp^2^ = 0.544) raises the possibility that caffeine’s neuromuscular effects on vertical jump are context-dependent, though this preliminary observation requires confirmation in adequately powered trials (*p* = 0.007, dz = 1.12). The specificity of caffeine’s effects on agility and reactive agility is consistent with evidence that adenosine receptor antagonism enhances neuromuscular drive and attentional resources underlying reactive motor responses [11,17]. The significantly lower cardiovascular load under caffeine (55.20 ± 4.84% vs. 58.49 ± 2.88%; *p* = 0.032, dz = 0.67), combined with the window-specific Testing Window × Supplementation interaction for %HRavg (F(2,24) = 5.88, *p* = 0.008, ηp^2^ = 0.329), indicates that caffeine-mediated cardiovascular economy may vary with the physiological context associated with menstrual-cycle timing. However, hormonal verification is required to confirm this interpretation. The ηp^2^ values reported here, with several exceeding 0.60, reflect both genuine experimental effects and systematic suppression of within-subject error variance in homogeneous elite athlete samples, inflating ηp^2^ relative to between-subjects benchmarks [1]. Cohen’s dz values, which are less susceptible to this design artifact, ranged from small (dz ≈ 0.67 for cardiovascular outcomes) to large (dz > 2.0 for fatigue effects on MAT), providing a complementary index of practical magnitude.

The integrative 3 × 2 × 2 × 2 factorial crossover design is a methodological strength, offering a within-subject characterization of multi-factor interactions rarely achieved in applied female sport research. However, several limitations constrain the interpretation of the findings. The modest sample (n = 13) provides adequate power for main effects but is insufficient for confirmatory interpretation of three-way and four-way interactions, which are designated exploratory a priori. Calendar-based cycle classification without LH testing or serum assays introduces classification uncertainty; all phase-related inferences must accordingly be bounded to testing window comparisons rather than endocrine states [1]. The absence of a blinding-effectiveness questionnaire means expectancy effects on perceptual outcomes (RPE, POMS, Hooper Index) cannot be excluded. A single familiarization session may have been insufficient to eliminate learning effects on the RAT and Stroop task, and no ICC data were collected to verify pre-experimental stability. Cardiovascular load was indexed via wearable HR monitoring, providing processed beat-to-beat estimates rather than raw ECG data; susceptibility to motion artifact during high-intensity volleyball actions, dependence on proprietary signal-processing algorithms, absence of a pre-exercise resting HR comparison, and inability to independently inspect raw waveforms represent constraints on the interpretation of %HRavg findings. Repeated administration of the Stroop task and RAT across 12 sessions introduces the possibility of residual practice effects; the single familiarization session may not have fully stabilized performance on high learning-curve tasks, and progressive performance improvements cannot be unambiguously attributed to experimental conditions in the absence of pre-experimental ICC and CV data. Because all participants were classified as intermediate chronotypes via the Horne–Ostberg questionnaire [19], the magnitude and direction of time-of-day effects on performance and caffeine responsiveness observed here may not generalize to strongly morning-type or evening-type athletes [8]. Without LH surge confirmation or serum progesterone assessment, the possibility of anovulatory cycles or luteal insufficiency during the study period cannot be excluded; participants classified as occupying the luteal testing window may not have been physiologically luteal in all cases, which would attenuate observed window differences [1,3]. Individual caffeine responder versus non-responder patterns were not examined; given known inter-individual variability in ergogenic caffeine responsiveness [12], future studies should consider responder stratification based on habitual caffeine intake and, where feasible, CYP1A2 genotype. Genotype-related variability in caffeine metabolism (CYP1A2 polymorphisms) and adenosine receptor sensitivity (ADORA2A polymorphisms) was not assessed; such genetic differences may partially account for inter-individual variation in ergogenic and perceptual responses and should be incorporated in future designs [12]. Repeated caffeine exposure across 12 sessions, despite the ≥32 h washout, may have progressively altered expectancy, caffeine habituation threshold, or adenosine receptor regulation in some participants; these temporal adaptation effects cannot be separated from experimental condition effects in the present design and should be addressed in future protocols through systematic randomization of supplementation conditions across the full session sequence [12]. Reliance on subjective sleep questionnaires rather than actigraphy introduces potential response bias, particularly under caffeine conditions where participants were aware of possible sleep disturbance; this represents a limitation given the study’s circadian emphasis. Among the principal findings, the main effects of testing window, caffeine, and time of day on ESS, POMS vigor and fatigue, Hooper Index, MAT, and Stroop reaction time are the most statistically robust, supported by large Bonferroni-corrected effect sizes; these represent the most reliable observations of the present study. By contrast, all three-way and four-way interaction terms are designated a priori as exploratory and are presented strictly for hypothesis generation. Cardiovascular and perceptual outcomes are additionally subject to wearable-device and expectancy confounds, respectively, and warrant particular interpretive caution.

### Practical Recommendations

The present data provisionally suggest that scheduling high-intensity training during the follicular testing window may be preferable; however, these observations require replication in larger samples with biochemical hormonal verification before operational recommendations can be established. Acute caffeine ingestion at 6 mg·kg^−1^, administered approximately 60 min before competition, represents a viable ergogenic strategy to reduce daytime sleepiness, attenuate fatigue-related perceptual strain, and support agility and cognitive performance, particularly in afternoon sessions and under post-match fatigue conditions. Given the higher incidence of insomnia complaints under caffeine, late-afternoon administration in athletes with compromised sleep capacity warrants caution. Individualized caffeine strategies incorporating cycle monitoring remain investigational and require replication in hormonally verified, adequately powered samples before prescriptive application [12,14].

## 5. Conclusions

The present study demonstrates that psychophysiological, cognitive, and neuromuscular performance in trained female volleyball players is independently influenced by calendar-based testing window, acute caffeine ingestion, time of day, and match-induced fatigue, with caffeine-related effects that varied in magnitude and specificity across outcome domains and contextual conditions. The follicular window consistently supported superior performance and well-being relative to the menstrual and luteal windows, while caffeine reliably improved alertness, mood, and agility-related outcomes. All higher-order interaction effects are designated exploratory, given the sample size of n = 13, and replication in larger cohorts with biochemical hormonal verification is necessary before definitive individualized prescriptions can be established. These findings nonetheless provide a methodologically integrative characterization of female athletes’ responses to caffeine within a circadian and menstrual-cycle framework, directly relevant to evidence-based performance preparation.

## Figures and Tables

**Table 1:** Countermovement Jump (CMJ), Modified Agility T-Test (MAT), and Reactive Agility Test (RAT) according to calendar-based testing window, supplementation condition, time of day, and fatigue state.

Variable	State	Menstrual/PLC		Menstrual/CAF		Follicular/PLC		Follicular/CAF		Luteal/PLC		Luteal/CAF	
		AM	PM	AM	PM	AM	PM	AM	PM	AM	PM	AM	PM
CMJ (cm)	Pre	30.85 ± 2.94	31.38 ± 2.90 †	31.42 ± 2.59	31.64 ± 2.17	32.02 ± 2.68 §	32.95 ± 2.35 †§	32.62 ± 3.24 §	34.17 ± 2.55 *†§	31.99 ± 2.76 ¶	33.25 ± 2.47 †¶	31.51 ± 3.84 ¶	32.95 ± 3.07 †¶
	Post	30.43 ± 2.86	30.35 ± 2.75 ‡	30.90 ± 2.67	31.13 ± 2.32	31.49 ± 2.53 ‡§	31.74 ± 2.56 ‡§	32.58 ± 3.07 *§	33.52 ± 2.75 *§	31.42 ± 2.70 ‡¶	31.98 ± 2.56 ‡¶	30.96 ± 3.77 ¶	32.45 ± 3.04 †¶
MAT (s)	Pre	6.80 ± 0.59	6.55 ± 0.53 †	6.48 ± 0.50	5.66 ± 0.41 *†	6.67 ± 0.67 §	6.25 ± 0.66 †§	5.63 ± 0.51 *§	4.88 ± 0.25 *†§	6.61 ± 0.60 ¶	6.30 ± 0.58 †¶	6.32 ± 0.23 ¶	5.78 ± 0.49 *†¶
	Post	6.82 ± 0.58	6.65 ± 0.55 ‡	6.37 ± 0.39	5.71 ± 0.46 *†	6.73 ± 0.65 §	6.41 ± 0.64 †‡§	5.59 ± 0.51 *‡§	4.92 ± 0.24 *†§	6.64 ± 0.60 ¶	6.44 ± 0.56 ‡¶	6.48 ± 0.32 ¶	5.91 ± 0.54 *†‡¶
RAT (s)	Pre	0.38 ± 0.10	0.35 ± 0.10 †	0.37 ± 0.09 *	0.34 ± 0.09 *†	0.22 ± 0.08 §	0.20 ± 0.08 †§	0.22 ± 0.07 §	0.20 ± 0.07 †§	0.38 ± 0.11 ¶	0.37 ± 0.11 †¶	0.37 ± 0.11 *¶	0.36 ± 0.11 *¶
	Post	0.38 ± 0.09	0.34 ± 0.10 †	0.37 ± 0.09	0.35 ± 0.10 †‡	0.22 ± 0.08 §	0.20 ± 0.08 †§	0.22 ± 0.07 ‡§	0.20 ± 0.07 †§	0.38 ± 0.11 ¶	0.35 ± 0.12 ¶	0.38 ± 0.10 ¶	0.36 ± 0.11 †¶

Values are mean ± SD; n = 13 per cell. CMJ: higher values indicate better performance. MAT and RAT: lower values indicate faster performance. * *p* < 0.05 vs. PLC within same window, time of day, and fatigue state. † *p* < 0.05 vs. AM within the same window, condition, and fatigue state. ‡ *p* < 0.05 vs. pre-fatigue state within same window, condition, and time of day. § *p* < 0.05 vs. menstrual window within the same condition, time of day, and fatigue state. ¶ *p* < 0.05 vs. follicular window within the same condition, time of day, and fatigue state. All post hoc comparisons were Bonferroni-adjusted.

**Table 2:** Profile of Mood States (POMS) according to calendar-based testing window, supplementation condition, time of day, and fatigue state.

Variable	State	Menstrual/PLC		Menstrual/CAF		Follicular/PLC		Follicular/CAF		Luteal/PLC		Luteal/CAF	
		AM	PM	AM	PM	AM	PM	AM	PM	AM	PM	AM	PM
Anxiety	Pre	7.92 ± 2.22	8.08 ± 1.80	7.15 ± 1.07	6.62 ± 1.04	7.54 ± 1.27	7.31 ± 1.25	7.00 ± 0.82	6.38 ± 0.65	7.85 ± 1.77	8.77 ± 2.52	7.08 ± 0.95	6.54 ± 0.97
	Post	7.62 ± 1.85 ‡	7.62 ± 1.26 ‡	6.85 ± 0.99 ‡	6.46 ± 0.88 ‡	7.31 ± 0.95 ‡	7.08 ± 0.86 ‡	6.69 ± 0.85 ‡	6.00 ± 0.71 ‡	7.62 ± 1.45 ‡	8.23 ± 2.09 ‡	6.77 ± 1.09 ‡	6.23 ± 1.09 ‡
Depression	Pre	10.77 ± 1.96	10.54 ± 2.07	10.15 ± 1.41	9.46 ± 1.05	9.23 ± 1.24	9.38 ± 0.77	7.92 ± 0.86	7.85 ± 0.69	8.85 ± 2.41	10.15 ± 2.15	10.31 ± 1.18	9.31 ± 1.55
	Post	10.31 ± 1.65	10.15 ± 1.21	9.77 ± 1.30	9.15 ± 0.80	8.92 ± 0.95	9.08 ± 0.86	7.69 ± 1.03	7.38 ± 0.65	8.46 ± 1.98	9.77 ± 1.88	9.54 ± 1.13	9.00 ± 1.53
Anger	Pre	12.15 ± 1.68	12.08 ± 1.44	10.23 ± 0.73	10.08 ± 0.95	10.62 ± 0.87	11.08 ± 0.76	9.77 ± 0.83	9.46 ± 0.97	10.46 ± 1.45	11.38 ± 1.66	10.15 ± 0.90	9.92 ± 1.04
	Post	11.69 ± 1.18 ‡	11.62 ± 1.04 ‡	10.00 ± 0.82 ‡	9.85 ± 1.14 ‡	10.23 ± 0.60 ‡	10.69 ± 0.85 ‡	9.38 ± 1.04 ‡	9.08 ± 0.86 ‡	10.08 ± 1.04 ‡	10.92 ± 1.26 ‡	9.85 ± 0.90 ‡	9.54 ± 1.05 ‡
Vigor	Pre	21.77 ± 1.36	21.62 ± 1.12	23.00 ± 1.00 *	23.23 ± 0.83 *	24.31 ± 0.95	24.62 ± 0.87	25.46 ± 0.88 *	26.00 ± 0.82 *	21.77 ± 1.54	21.46 ± 1.27	23.08 ± 1.04 *	23.15 ± 0.80 *
	Post	22.54 ± 1.27 ‡	22.46 ± 0.88 ‡	23.31 ± 0.95 *‡	23.69 ± 1.03 *‡	25.15 ± 1.07 ‡	25.62 ± 1.04 ‡	25.85 ± 0.99 *‡	26.46 ± 0.66 *‡	22.62 ± 1.50 ‡	22.23 ± 1.09 ‡	23.46 ± 1.13 *‡	23.54 ± 0.88 *‡
Fatigue	Pre	6.62 ± 1.71	6.54 ± 1.45	6.08 ± 0.86 *	5.92 ± 0.86 *	6.23 ± 0.83	6.15 ± 0.69	5.69 ± 0.63 *	5.46 ± 0.52 *	7.62 ± 1.89	7.08 ± 0.95	6.08 ± 0.76 *	5.85 ± 0.69 *
	Post	6.38 ± 1.26 ‡	6.31 ± 1.18 ‡	5.92 ± 0.76 *‡	5.69 ± 0.63 *‡	6.08 ± 0.76 ‡	5.92 ± 0.64 ‡	5.38 ± 0.51 *‡	5.23 ± 0.44 *‡	7.31 ± 1.55 ‡	6.77 ± 0.73 ‡	5.69 ± 0.75 *‡	5.46 ± 0.52 *‡
Confusion	Pre	6.62 ± 1.26	6.54 ± 1.33	6.23 ± 0.83	6.00 ± 0.82	5.92 ± 0.64	5.85 ± 0.38	5.46 ± 0.52	5.46 ± 0.52	7.54 ± 1.66	6.46 ± 1.13	6.15 ± 0.69 *	6.08 ± 0.86 *
	Post	6.54 ± 1.13 ‡	6.46 ± 1.20 ‡	5.85 ± 0.69	5.62 ± 0.65	5.85 ± 0.55 ‡	5.77 ± 0.44 ‡	5.23 ± 0.44	5.23 ± 0.44	7.46 ± 1.51 ‡	6.46 ± 1.13 ‡	5.85 ± 0.69 *	5.62 ± 0.65 *
TMD	Pre	22.23 ± 9.10	22.15 ± 8.25	16.85 ± 4.38 §	15.00 ± 3.34 §	15.23 ± 2.92 §	15.08 ± 2.02 §	9.62 ± 2.18 §	8.62 ± 1.71 §	22.46 ± 8.39	22.38 ± 8.30	15.15 ± 2.82 ¶	15.31 ± 3.01 ¶
	Post	20.00 ± 6.89	19.69 ± 5.07	15.08 ± 3.23 §	13.08 ± 3.33 §	13.23 ± 2.35 §	12.92 ± 1.98 §	7.69 ± 2.50 §	6.46 ± 1.13 §	18.31 ± 6.50	19.92 ± 6.50	14.23 ± 3.35 ¶	12.31 ± 3.09 ¶

Values are mean ± SD; n = 13 per cell. Higher scores indicate greater intensity for all negative subscales (anxiety, depression, anger, fatigue, confusion); higher vigor scores indicate better affective readiness. TMD = Total Mood Disturbance. * *p* < 0.05 vs. PLC within same window, time of day, and fatigue state. ‡ *p* < 0.05 vs. pre-fatigue state within same window, condition, and time of day. § *p* < 0.05 vs. menstrual window within same condition, time of day, and fatigue state. ¶ *p* < 0.05 vs. follicular window within same condition, time of day, and fatigue state. All post hoc comparisons were Bonferroni-adjusted.

**Table 3:** Hooper Index according to calendar-based testing window, supplementation condition, time of day, and fatigue state.

Fatigue State	Menstrual/PLC		Menstrual/CAF		Follicular/PLC		Follicular/CAF		Luteal/PLC		Luteal/CAF	
	AM	PM	AM	PM	AM	PM	AM	PM	AM	PM	AM	PM
Pre-fatigue	16.23 ± 2.13	14.46 ± 1.81 †	15.62 ± 1.33 *	13.92 ± 1.32 *†	11.85 ± 1.07 §	10.54 ± 1.13 *†§	11.38 ± 0.77 §	10.15 ± 0.69 *†§	15.46 ± 1.76 ¶	13.54 ± 1.13 †¶	14.92 ± 0.86 *¶	13.08 ± 0.64 *†¶
Post-fatigue	15.08 ± 2.02 ‡	13.15 ± 1.63 †‡	14.54 ± 1.51 *‡	12.69 ± 1.18 *†‡	11.08 ± 1.12 ‡§	9.31 ± 1.60 *†‡§	10.77 ± 0.83 ‡§	9.08 ± 1.38 *†‡§	14.31 ± 1.70 ‡¶	12.46 ± 0.88 †‡¶	13.85 ± 0.99 *‡¶	12.08 ± 0.64 *†‡¶

Values are mean ± SD; n = 13 per cell. Scored 4–28; higher values indicate greater perceived psychophysiological strain. * *p* < 0.05 vs. PLC within the same window, time of day, and fatigue state. † *p* < 0.05 vs. AM within the same window, condition, and fatigue state. ‡ *p* < 0.05 vs. pre-fatigue state within the same window, condition, and time of day. § *p* < 0.05 vs. menstrual window within the same condition, time of day, and fatigue state. ¶ *p* < 0.05 vs. follicular window within the same condition, time of day, and fatigue state. All comparisons were Bonferroni-adjusted.

**Table 4:** Cardiovascular load (%HRavg) and rating of perceived exertion (RPE) according to calendar-based testing window, supplementation condition, and time of day.

Variable	Menstrual/PLC		Menstrual/CAF		Follicular/PLC		Follicular/CAF		Luteal/PLC		Luteal/CAF	
	AM	PM	AM	PM	AM	PM	AM	PM	AM	PM	AM	PM
**%HRavg (%)**	58.66 ± 5.71	56.32 ± 8.01 †	62.25 ± 5.17	58.40 ± 6.92 †	58.51 ± 6.27	56.84 ± 7.09 †	54.65 ± 9.05	49.11 ± 9.21 *†	60.96 ± 4.41	59.65 ± 7.68 †	54.17 ± 9.20 *	52.61 ± 8.47 *†
**RPE (AU)**	15.46 ± 1.13	15.69 ± 0.75	15.08 ± 0.95 *	14.92 ± 0.64 *	15.38 ± 0.77 §	15.69 ± 0.75 §	15.62 ± 0.96 §	16.15 ± 0.69 §	15.31 ± 0.95 ¶	15.46 ± 0.66 ¶	15.08 ± 0.86 ¶	15.15 ± 0.90 ¶

Values are mean ± SD; n = 13 per cell. %HRavg = mean heart rate expressed as a percentage of estimated maximum heart rate (220 − age). RPE = rating of perceived exertion assessed with the Borg RPE scale (6–20). * *p* < 0.05 vs. PLC within the same window and time-of-day condition. † *p* < 0.05 vs. AM within the same window and supplementation condition. § *p* < 0.05 vs. menstrual window within the same condition and time of day. ¶ *p* < 0.05 vs. follicular window within the same condition and time of day. All comparisons were Bonferroni-adjusted.

**Table 5:** Sleep-related and alertness outcomes according to calendar-based testing window and supplementation condition.

Panel A. Epworth Sleepiness Scale (ESS; range 0–24; higher scores indicate greater daytime sleepiness)												
	Menstrual Window				Follicular Window				Luteal Window			
**ESS**	**PLC-AM**	**PLC-PM**	**CAF-AM**	**CAF-PM**	**PLC-AM**	**PLC-PM**	CAF-AM	CAF-PM	PLC-AM	PLC-PM	CAF-AM	CAF-PM
	8.23 ± 1.09	7.85 ± 1.07 †	7.92 ± 0.86 *	7.46 ± 0.97 *†	5.92 ± 0.76 §	5.23 ± 0.73 †§	5.77 ± 0.73 *§	5.08 ± 0.76 *†§	7.23 ± 1.01 §¶	6.85 ± 0.99 †§¶	7.00 ± 0.82 *§¶	6.46 ± 0.97 *†§¶
**Panel B. Pittsburgh Sleep Quality Index (PSQI; range 0–21; higher = poorer sleep quality) and Spiegel Sleep Questionnaire (range 0–30; higher = better sleep quality)** Time-of-day factor not included in these models; values represent session-pooled means per window and condition.												
**Variable**		**Menstrual PLC**		**Menstrual CAF**		**Follicular PLC**	**Follicular CAF**		**Luteal PLC**		**Luteal CAF**	
PSQI		6.38 ± 0.96 ᵃ		6.08 ± 0.76 ᵃ		4.08 ± 0.86 ᵇ	4.00 ± 0.58 ᵇ		5.92 ± 1.66 ᵃ		6.00 ± 1.00 ᵃ	
Spiegel		20.08 ± 1.26 ᵇ		20.38 ± 1.04 *ᵇ		20.54 ± 1.05 ᵇ	20.92 ± 1.04 *ᵇ		24.15 ± 1.21 ᵃ		24.54 ± 0.78 *ᵃ	

Values are mean ± SD; n = 13 per cell. CAF = caffeine; PLC = placebo; AM = morning session (08:00 h); PM = afternoon session (18:00 h). ᵃ *p* < 0.05 vs. follicular window within same supplementation condition (Bonferroni-adjusted). ᵇ *p* < 0.05 vs. menstrual and luteal windows within the same supplementation condition (Bonferroni-adjusted). * *p* < 0.05 vs. PLC within the same window and time-of-day condition. † *p* < 0.05 vs. AM within the same window and supplementation condition. § *p* < 0.05 vs. menstrual window within the same condition and time of day. ¶ *p* < 0.05 vs. follicular window within the same condition and time of day. All post hoc comparisons were Bonferroni-adjusted.

**Table 6:** Stroop task reaction time (s) and error count (n) according to calendar-based testing window, supplementation condition, time of day, and fatigue state.

Panel A. Reaction Time (seconds)													
Plate	State	Menstrual/PLC		Menstrual/CAF		Follicular/PLC		Follicular/CAF		Luteal/PLC		Luteal/CAF	
		AM	PM	AM	PM	AM	PM	AM	PM	AM	PM	AM	PM
P1 Color	Pre	11.48 ± 1.51	11.11 ± 1.49	10.76 ± 1.09	10.39 ± 1.13 *†	10.08 ± 1.04 §	10.26 ± 1.24 §	9.71 ± 0.76 §	9.66 ± 0.86 §	11.47 ± 1.48 ¶	11.17 ± 1.41 ¶	10.57 ± 1.14 *¶	10.39 ± 1.02 *¶
	Post	11.48 ± 1.52	11.13 ± 1.48	10.77 ± 1.08	10.42 ± 1.12 *†	10.04 ± 1.05 §	10.17 ± 1.16 §	9.74 ± 0.78 §	9.65 ± 0.81 §	11.48 ± 1.48 ¶	11.18 ± 1.52 ¶	10.58 ± 1.19 ¶	10.41 ± 1.02 *¶
P2 Word	Pre	14.05 ± 1.55	14.09 ± 2.76	13.71 ± 1.21	13.40 ± 1.74	12.51 ± 2.99 §	11.49 ± 2.48 †§	12.18 ± 2.78 §	11.23 ± 2.85 †§	14.25 ± 3.24 ¶	13.39 ± 3.29 †¶	13.98 ± 2.38 ¶	13.07 ± 1.68 ¶
	Post	14.77 ± 2.73	13.92 ± 2.66 †	14.15 ± 2.02	13.41 ± 1.75 †	12.62 ± 3.44 §	11.55 ± 2.52 §	12.15 ± 2.99 §	11.32 ± 3.12 †§	14.15 ± 3.09 ¶	12.92 ± 2.17 ¶	14.00 ± 2.35 ¶	13.09 ± 1.71 ¶
P3 Interf.	Pre	16.93 ± 4.03	16.32 ± 4.26 †	15.93 ± 2.74	15.31 ± 2.87	14.57 ± 3.61 §	13.87 ± 3.53 †§	13.94 ± 2.38 §	13.30 ± 3.14 §	16.64 ± 3.87 ¶	15.91 ± 3.85 †¶	15.61 ± 3.83 ¶	15.31 ± 3.28 ¶
	Post	16.61 ± 4.15	16.16 ± 4.12	15.94 ± 2.73	15.33 ± 2.86	14.50 ± 3.38 §	13.68 ± 3.52 †§	14.01 ± 2.33 §	13.32 ± 3.11 §	16.62 ± 3.86 ¶	15.60 ± 3.53 †¶	15.63 ± 3.83 ¶	15.32 ± 3.28 ¶
**Panel B. Errors (count)**													
Plate	**State**	**Menstrual/PLC**		**Menstrual/CAF**		**Follicular/PLC**		**Follicular/CAF**		**Luteal/PLC**		**Luteal/CAF**	
		**AM**	**PM**	**AM**	**PM**	**AM**	**PM**	**AM**	**PM**	**AM**	**PM**	**AM**	**PM**
P1	Pre	2.00 ± 0.71	1.00 ± 0.82 †	1.15 ± 0.55	0.77 ± 0.44	0.92 ± 0.86 §	0.46 ± 0.52 †§	0.85 ± 0.38 §	0.31 ± 0.48 †§	1.85 ± 0.80 ¶	0.85 ± 0.69 †¶	1.46 ± 0.52 ¶	0.69 ± 0.48 †¶
	Post	1.23 ± 0.73	0.92 ± 0.64	1.00 ± 0.58	0.77 ± 0.44	0.92 ± 0.76 §	0.54 ± 0.66 §	0.77 ± 0.44 §	0.38 ± 0.51 §	1.46 ± 1.39 ¶	1.08 ± 0.64 ¶	1.38 ± 0.65 ¶	0.77 ± 0.44 ¶
P2	Pre	1.85 ± 1.14	1.23 ± 0.73	1.69 ± 0.75	1.15 ± 0.55	1.08 ± 0.76 §	0.31 ± 0.48 †§	0.85 ± 0.55 §	0.23 ± 0.44 †§	2.31 ± 0.95 ¶	1.62 ± 0.96 ¶	1.54 ± 0.52 *¶	1.08 ± 0.76 ¶
	Post	1.85 ± 0.69	1.15 ± 0.99	1.77 ± 0.60	1.23 ± 0.60	0.85 ± 0.55 §	0.15 ± 0.38 †§	0.77 ± 0.44 §	0.15 ± 0.38 †§	1.92 ± 1.38 ¶	1.46 ± 0.88 ¶	1.62 ± 0.51 ¶	1.15 ± 0.38 ¶
P3	Pre	3.08 ± 1.61	1.85 ± 1.63†	2.85 ± 0.80	2.00 ± 0.91 †	1.00 ± 0.82 §	0.15 ± 0.38 †§	0.92 ± 0.64 §	0.38 ± 0.51 †§	3.00 ± 1.58 ¶	2.15 ± 1.41 †¶	2.62 ± 1.61 ¶	2.23 ± 1.01 ¶
	Post	2.15 ± 1.21	1.38 ± 1.04†	2.77 ± 1.01	1.92 ± 0.76 †	0.77 ± 0.60 §	0.54 ± 0.52 †§	0.85 ± 0.38 §	0.46 ± 0.52 §	2.92 ± 1.32 ¶	2.38 ± 1.39 ¶	2.85 ± 1.07 ¶	2.31 ± 0.95 ¶

P1 = Plate 1 (color naming); P2 = Plate 2 (word reading); P3 = Plate 3 (interference condition). * *p* < 0.05 vs. PLC within same window, time of day, and fatigue state. † *p* < 0.05 vs. AM within the same window, condition, and fatigue state. § *p* < 0.05 vs. menstrual window within the same condition, time of day, and fatigue state. ¶ *p* < 0.05 vs. follicular window within the same condition, time of day, and fatigue state. All comparisons were Bonferroni-adjusted.

## Data Availability

The data supporting the findings of this study are available from the corresponding authors upon reasonable request.
